# Maize ZmBES1/BZR1-5 Decreases ABA Sensitivity and Confers Tolerance to Osmotic Stress in Transgenic *Arabidopsis*

**DOI:** 10.3390/ijms21030996

**Published:** 2020-02-03

**Authors:** Fuai Sun, Haoqiang Yu, Jingtao Qu, Yang Cao, Lei Ding, Wenqi Feng, Muhammad Hayder Bin Khalid, Wanchen Li, Fengling Fu

**Affiliations:** Key Laboratory of Biology and Genetic Improvement of Maize in Southwest Region, Ministry of Agriculture, Maize Research Institute, Sichuan Agricultural University, Chengdu 611130, China; sunfuai@stu.sicau.edu.cn (F.S.); qujingtao@sicau.edu.cn (J.Q.); caoy@stu.sicau.edu.cn (Y.C.); dinglei@stu.sicau.edu.cn (L.D.); fwq@stu.sicau.edu.cn (W.F.); haider2323@gmail.com (M.H.B.K.); aumdyms@sicau.edu.cn (W.L.)

**Keywords:** maize, BES1/BZR1, abiotic stress, ABA, RNA-seq

## Abstract

The BRI1-EMS suppressor 1 (BES1)/brassinazole-resistant 1 (BZR1) transcription factors, key components in the brassinosteroid signaling pathway, play pivotal roles in plant growth and development. However, the function of BES1/BZR1 in crops during stress response remains poorly understood. In the present study, we characterized ZmBES1/BZR1-5 from maize, which was localized to the nucleus and was responsive to abscisic acid (ABA), salt and drought stresses. Heterologous expression of *ZmBES1/BZR1-5* in transgenic *Arabidopsis* resulted in decreased ABA sensitivity, facilitated shoot growth and root development, and enhanced salt and drought tolerance with lower malondialdehyde (MDA) content and relative electrolyte leakage (REL) under osmotic stress. The RNA sequencing (RNA-seq) analysis revealed that 84 common differentially expressed genes (DEGs) were regulated by ZmBES1/BZR1-5 in transgenic *Arabidopsis*. Subsequently, gene ontology and KEGG pathway enrichment analyses showed that the DEGs were enriched in response to stress, secondary metabolism and metabolic pathways. Furthermore, 30 DEGs were assigned to stress response and possessed 2–15 E-box elements in their promoters, which could be potentially recognized and bound by ZmBES1/BZR1-5. Taken together, our results reveal that the ZmBES1/BZR1-5 transcription factor positively regulates salt and drought tolerance by binding to E-box to induce the expression of downstream stress-related genes. Therefore, our study contributes to the better understanding of BES1/BZR1 function in the stress response of plants.

## 1. Introduction

Environmental stimuli significantly restrict plant growth, development and reproduction. Among the various stresses, high salinity and drought are two major adverse abiotic factors that occur frequently and simultaneously, reducing plant productivity [1]. Under salt and drought stresses, plants undergo water deprivation and experience excess Na^+^ and Cl^−^ uptake, which results in cellular disruption and eventually death [2]. To cope with these challenges, plants have evolved multifaceted adaption networks associated with changes in morphology, physiology and photosynthesis to promote their chances of survival [3,4,5,6]. For instance, as the root is the first defensive line for soilborne stresses, including water deficits and high salt, plants can resist these stresses by adjusting their root system architecture [7,8]. Moreover, phytohormones and transcription factors regulate the expression of stress-induced genes, and the resulting signal transduction cascade can help plants survive under adverse conditions [9,10].

Brassinosteroids (BRs) are plant-specific steroidal hormones that have crucial roles in plant growth, development and stress responses [11,12,13,14]. BRs are perceived by receptor kinase complexes, including BRASSINOSTEROID INSENSITIVE1 (BRI1), BRI1-ASSOCIATED RECEPTOR KINASE1 (BAK1), or leucine-rich repeat receptor-like kinases (RLKs) on the plasma membrane [15,16,17]. After perceiving BRs, these kinases are activated and initiate an intracellular signaling cascade, including dephosphorylation of BRASSINOSTEROID INSENSITIVE 2 (BIN2), resulting in inactivation of BIN2. The inactivated BIN2 cannot phosphorylate BRI1 EMS SUPPRESSOR 1 (BES1) and its homolog BRASSINAZOLE RESISTANT 1 (BZR1), which induces the accumulation of non-phosphorylated forms of BES1 and BZR1 in the nucleus [17,18]. Thereafter, the activated BES1 or BZR1 binds to the E-box (CANNTG) element enriched in BR-induced genes or BRRE (CGTGT/CG) abundant in BR-repressed genes, to regulate plant growth and BR synthesis [18,19,20,21,22].

BES1 shares a high identity with BZR1 at the amino acid level (88%) and N-terminal domain (97%), and several family members have been identified, including BES1/BZR1 homologs 1–4 (BEH1–4) [18,19]. These transcription factors show functional redundancy in the BR signaling transduction pathway and, hence, have been renamed BES1/BZR1s [18,19,23]. It has been demonstrated that BES1/BZR1 family members are plant-specific transcription factors and play essential roles in plant growth and development [24,25]. Previous studies have shown that BES1/BZR1s regulate root elongation, flowering and seed germination through regulating the expression of the downstream target genes in the BR signaling pathway [24,26,27]. Likewise, BES1/BZR1s control cell and hypocotyl elongation by interacting with the light signal regulators PIF4, PhyB, UVB8 and CRY1 [20,28,29,30]. Recently, BES1/BZR1s have been reported to regulate drought, heat and freezing stress response through regulating the expression of *glutathione S-transferase 1* (*GST1*), *RESPONSIVE TO DESICCATION 26* (*RD26*) and *CBF* genes, as well as interacting with RD26 and WRKY transcription factors [31,32,33,34,35]. In addition to their crucial roles in abiotic stress, BES1/BZR1s also function in response to biotic and nutrition stresses, such as immunity, autophagy, nitrogen and phosphorus starvation [36,37,38]. However, the function of BES1/BZR1s in crops remains obscure.

Maize is an important cereal crop globally, and possesses a complex genome, which might lead to evolutional and functional diversification among the *ZmBES1/BZR1* genes [39]. However, the function of *ZmBES1/BZR1* genes remains unknown. In our previous study, the *ZmBES1/BZR1*-*5* gene was characterized and showed structural and evolutionary diversity compared to other *ZmBES1/BZR1* genes in maize [23]. In order to study the function of the *ZmBES1/BZR1*-*5* gene, in this study, the expression pattern and subcellular localization of ZmBES1/BZR1-5 were analyzed. Subsequently, the *ZmBES1/BZR1-5* gene was transformed into *Arabidopsis* to evaluate its function. We found that the ZmBES1/BZR1-5 decreased abscisic acid (ABA) sensitivity and enhanced tolerance to osmotic stress in transgenic *Arabidopsis*. Our novel findings provide additional knowledge for better understanding the function of *ZmBES1/BZR1s* in maize.

## 2. Results

### 2.1. Expression Analysis of ZmBES1/BZR1-5 Gene

**Figure 1:**
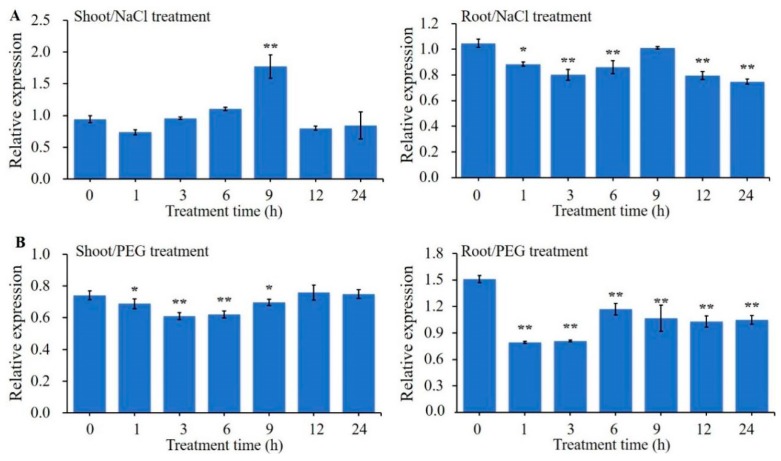
Expression patterns of *ZmBES1/BZR1-5* from qRT-PCR. The three-leaf stage of B73 seedlings were exposed to a water solution supplemented with 250mM NaCl (**A**) and 16% PEG-6000 (**B**). All values are means (±SE) of three biological replicates. The *ZmGAPDH* gene was used as internal reference. The relative expression level was calculated and normalized using the 2^−ΔΔCT^ method of the CFX Manger™ software version 2.0 (Bio-Rad, Berkeley, CA, USA). * *p* < 0.05 and ** *p* < 0.01 by Student’s *t* test, respectively.

The expression patterns of genes are often indicative of their potential function. Hence, in this study, the expression patterns of the *ZmBES1/BZR1-5* gene under osmotic stresses, including NaCl and PEG-6000 treatments, were analyzed by quantitative real time PCR (qRT-PCR). The results of qRT-PCR showed that the expression of *ZmBES1/BZR1-5* in maize shoot was induced by NaCl treatment and reached a peak after 9 h of treatment (Figure 1A). However, in contrast to shoot samples, the expression of *ZmBES1/BZR1-5* in maize root was significantly inhibited by NaCl treatment and reached a minimum after 24 h of treatment (Figure 1A). Moreover, the expression of *ZmBES1/BZR1-5* in maize shoot and root was inhibited by PEG treatment and both reached a minimum at 3 h after treatment (Figure 1B). In our previous study, we demonstrated that the expression of the *ZmBES1/BZR1-5* gene was upregulated in maize shoots and downregulated in roots in response to ABA [23]. These results indicate that *ZmBES1/BZR1-5* may play an important role in stress tolerance to salt, drought and ABA response.

### 2.2. Subcellular Localization of ZmBES1/BZR1-5

**Figure 2:**
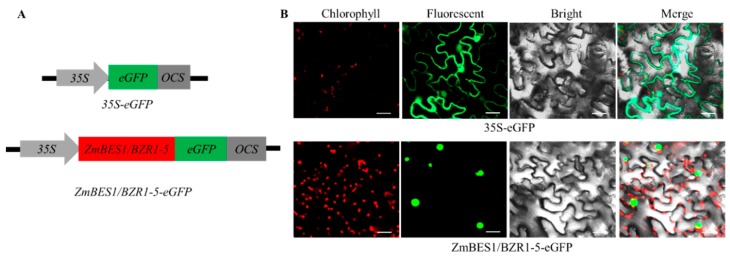
Subcellular localization of ZmBES1/BZR1-5 in tobacco leaf epidermal cells. (**A**) The diagram of the vector for transient expression. (**B**) GFP fluorescence observed by confocal microscopy. Scale bars = 20 µm.

As plant-specific transcription factors, BES1/BZR1s accumulate in the nucleus to regulate the expression of downstream genes. Therefore, to determine the subcellular localization of the ZmBES1/BZR1-5 protein, a recombinant plasmid expressing *ZmBES1/BZR1-5* without a stop codon fused to an enhanced green fluorescent protein gene (*eGFP*), named *35S*-*ZmBES1*/*BZR1-5*-*eGFP*. The construct was transfected into tobacco leaf epidermal cells, with the *35S*-*eGFP* vector transfected as a control. The results of confocal imaging showed that GFP fluorescence could be observed in both the cytoplasm and nucleus of tobacco leaves transformed with *35S*-*eGFP*, but only the nucleus of tobacco leaves transformed with *35S*-*ZmBES1/BZR1-5*-*eGFP*. The results suggest that ZmBES1/BZR1-5 functions in the nucleus (Figure 2).

### 2.3. ZmBES1/BZR1-5 Facilitates Growth Promotion, and Reduces ABA Sensitivity in Arabidopsis

**Figure 3:**
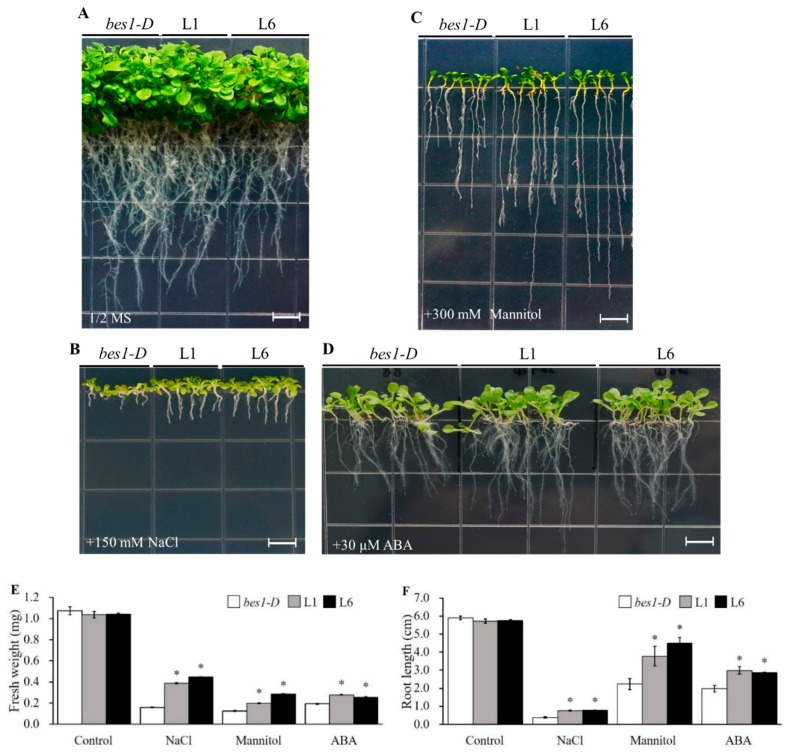
Phenotypes of *bes1-D*, L1 and L6 lines under optimal conditions (**A**), 150mM NaCl (**B**), 300 mM mannitol (**C**) and 30 μM abscisic acid (ABA) (**D**). Quantification of fresh weight (**E**) and root length (**F**) of every line grown on 1/2× MS medium plates for three weeks. All values are means (±SE) of three biological replicates. *bes1-D*, L1 and L6 represent either an untransformed mutant or two homozygous T_3_ lines. * represents *p* < 0.05 by Student’s *t* test. Scale bars = 0.5 cm.

To evaluate the function of *ZmBES1/BZR1-5*, we generated transgenic *Arabidopsis BES1* mutants harboring *ZmBES1/BZR1-5* (Figure S1). The untransformed *bes1-D* mutant, L1 and L6 lines were planted on 1/2× MS media plates supplemented with 150 mM NaCl, 300 mM mannitol or 30 μM ABA. Subsequently, the root length and fresh weight were measured for each line. As shown in Figure 3, on the 1/2× MS plates without treatments (control), all plants exhibited a vigorous phenotype, and the root length and fresh weight showed no significant difference among these lines. However, on the 1/2× MS plates with NaCl, mannitol or ABA, the root length and fresh weight of the L1 and L6 lines was significantly longer or larger compared to the *bes1-D* mutant. The results indicate that the expression of the *ZmBES1/BZR1-5* gene accelerates growth and root development under osmotic stress, and decreases ABA sensitivity in transgenic *Arabidopsis*.

### 2.4. ZmBES1/BZR1-5 Enhances Salt and Drought Tolerance in Arabidopsis

**Figure 4:**
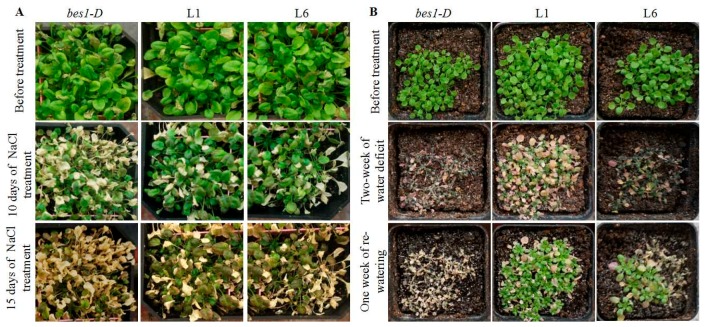
(**A**) Phenotypes of the *bes1-D* mutant, L1 and L6 lines under salt stress. Three-week-old seedlings were subjected to 250 mM NaCl solution twice, with an interval of 3 days between exposures. (**B**) Phenotypes of the *bes1-D* mutant, L1 and L6 lines under drought stress. Two-week-old seedlings were kept under water deficit conditions for two weeks, then re-watered and kept under optimal conditions for one week.

For further phenotyping of the *bes1-D* mutant, as well as the L1 and L6 lines under osmotic stress, the seedlings on soil were treated with salt and drought stresses. In the salt stress experiment, three-week-old seedlings were treated with a 250 mM NaCl solution twice, with an interval of 3 days between exposures. As shown in Figure 4A, all plants grew normally before stress, but after NaCl stress, the *bes1-D* plants exhibited wilting symptoms on the tenth day of stress and died by the fifteenth day of stress. However, the L1 and L6 lines showed a more vigorous phenotype and less severe wilting compared to *bes1-D*. 

Similarly, in the drought stress experiment, plants were grown under normal watering conditions for two weeks, then used for withholding water. After two weeks of water deprivation, the growth of the *bes1-D* mutants was severely inhibited, but L1 and L6 lines showed a vigorous phenotype. Subsequently, after re-watering for one week, the L1 and L6 lines were recovered, while the *bes1-D* mutants had died (Figure 4B). 

**Figure 5:**
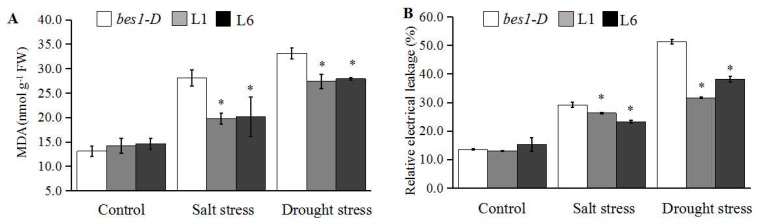
Quantification of MDA content (**A**) and REL (**B**) of the *bes1-D* mutant, L1 and L6 lines after salt and drought stress. For salt treatment, three-week-old seedlings were watered with 250 mM NaCl solution for 3 days, then used to measurement MDA and REL. For the drought treatment, two-week-old seedlings were kept under water deficit conditions for 7 days, then used to measure MDA and REL. All values are means (±SE) of three biological replicates. * *p* < 0.05 by Student’s *t* test.

Malondialdehyde (MDA) content and relative electrolyte leakage (REL) are widely used as indicators when evaluating plant tolerance to abiotic stresses. Here, before salt and drought stress, there was no significant difference (*p* > 0.05) in MDA content and REL between the *bes1-D* mutant and transgenic lines. However, after treatment, the MDA content and REL of L1 and L6 lines was significantly lower (*p* > 0.05) compared to the *bes1-D* mutant, although the MDA content and REL of all plants increased after salt and drought treatment (Figure 5).

The results indicate that the transgenic lines are more tolerant to osmotic stress compared to the *bes1-D* mutant.

### 2.5. ZmBES1/BZR1-5 Regulates the Expression of Stress-Related Genes

**Figure 6:**
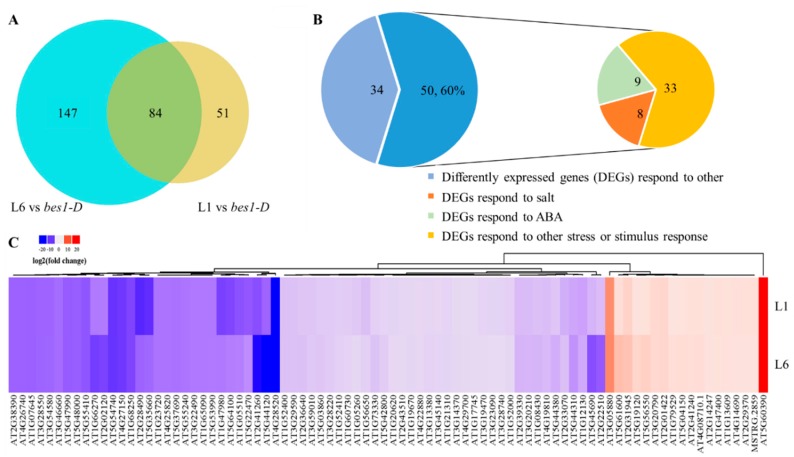
Identification of differently expressed genes (DEGs) in the L1 and L6 lines compared with the untransformed *bes1-D* mutant. (**A**) Venn diagram of DEGs. (**B**) The category of DEGs. (**C**) Heatmap of DEGs.

**Figure 7:**
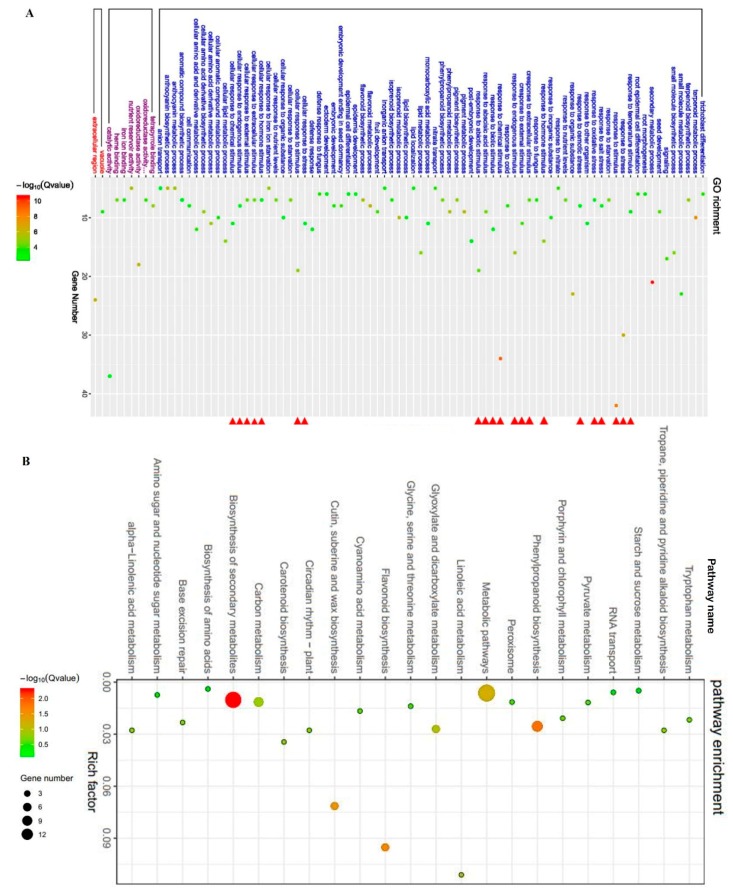
Gene ontology (GO) analysis (**A**) and KEGG enrichment analysis (**B**) of common DEGs in the L1 and L6 lines compared to the *bes1-D* mutant. The red triangle in Panel A indicates the stress or stimulus response.

RNA sequencing (RNA-seq) is widely used to identify stress-related genes, and can also provide key information regarding the expression of genes regulated by transcription factors [40,41]. To explore the genes regulated by ZmBES1/BZR1-5, we performed RNA-seq using the Illumina HiSeq3000 system. As shown in Figure 6, 135 and 231 differentially expressed genes (DEGs) were identified in the L1 and L6 lines as compared to *bes1-D*, respectively, using the threshold of a two-fold change and *p* < 0.05 to indicate significance as determined using a Student’s *t* test. Among these genes, 84 DEGs were shared by the L1 and L6 lines. Furthermore, 66 DEGs were downregulated and 18 DEGs were upregulated in the transgenic lines. The results of gene ontology (GO) term analyses showed that the 50 common genes that accounted for 59.52% of all the DEGs were associated with stress or stimulus response (Figure 6 and Figure 7A, Table S1). In total, 30 DEGs were directly annotated with a response to stress in the transgenic lines, in which 6 DEGs were significantly induced and 24 DEGs were significantly inhibited. Notably, 8 genes were responsive to salt and osmotic stresses and 9 DEGs responded to ABA (Figure 6 and Table S1). Among them, expression of the rare cold-inducible 2a gene (*RCI2A*, AT3G05880) was significantly upregulated by ZmBES1/BZR1-5, which contributes to improved salt tolerance [42,43]. The KEGG enrichment analysis indicated that 84 DEGs participate in secondary metabolism or metabolic pathways (Figure 7B).

**Figure 8:**
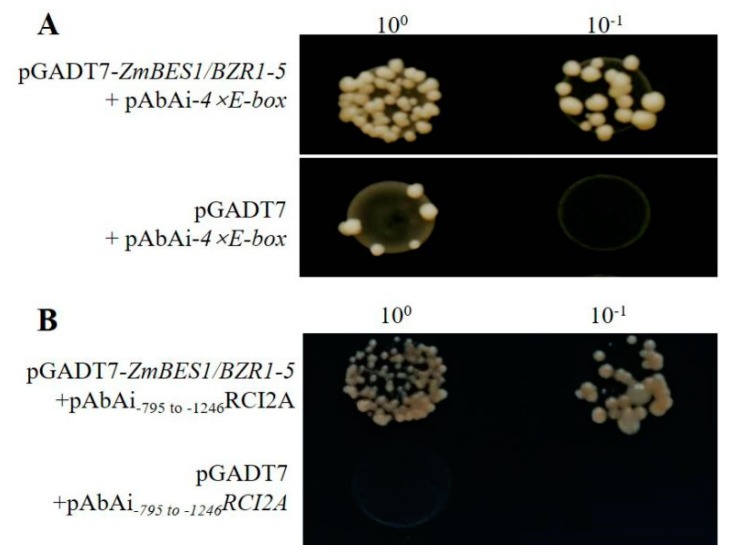
DNA binding assay using Y1H. (**A**) Y1H between the E-box and ZmBES1/BZR1-5. (**B**) Y1H between the −795 to −1246 promoter of the *RCI2A* gene and ZmBES1/BZR1-5.

To identify the potential target genes regulated by ZmBES1/BZR1-5, the 2000 bp upstream of DEGs were retrieved using the sequence data in the TAIR10 genome release and used to search for E-box or BRRE elements. The results of the promoter analysis showed there were abundant E-box elements (2–15) in the promoter regions of the 30 stress-related DEGs (Table S2), indicating that ZmBES1/BZR1-5 might bind to these E-box elements to regulate their expression. Hence, a yeast-one hybrid (Y1H) assay was performed to test whether ZmBES1/BZR1-5 binds to the E-box elements. As shown in Figure 8A, the yeast harboring pAbAi−4×E-box and pGADT7–*ZmBES1/BZR1-5* grew well on the SD/−Leu plate containing 400 ng/ml aureobasidin A (AbA) to screen for positive colonies, suggesting that ZmBES1/BZR1-5 could bind to the E-box element directly. Furthermore, the −795 to −1246 bp promoter region of the *RCI2A* gene containing seven E-box elements was amplified and used in Y1H experiments to detect whether ZmBES1/BZR1-5 could bind to the *RCI2A* promoter. The results show that the yeast with pAbAi_−795_ to _−1246_*RCI2A* and pGADT7–*ZmBES1/BZR1-5* grew well on the SD/−Leu plate with 400 ng/ml AbA (Figure 8B), indicating that ZmBES1/BZR1-5 bound to the *RCI2A* promoter. Hence, the above results suggest that these DEGs could be target genes of the ZmBES1/BZR1-5 transcription factor.

## 3. Discussion

The BES1/BZR1s are a large family of plant-specific transcription factors that are involved in plant growth and developmental processes, autophagy as well as response to nitrogen starvation [18,19,38]. Some prior studies have shown that the *BES1/BZR1* genes are regulated by drought, salt, cold and heat stress in *Brassica rapa*, *Eucalyptus grandis*, *Brassica napus* and *Arabidopsis* [44,45,46], and accelerate drought, heat and freezing tolerance in tomato or *Arabidopsis* [32,33,34,35], implying that *BES1/BZR1* genes participate in the osmotic stress response. In our study, the *ZmBES1/BZR1-5* gene also responds to salt and drought stress in maize (Figure 1), which may be related to the *cis*-acting elements of its promoter [23]. The ZmBES1/BZR1-5 protein was likewise found to be localized to the nucleus in tobacco leaf and in transgenic *Arabidopsis* (Figure 2 and Figure S1B), and did not generate any defective phenotype in transgenic *Arabidopsis* (Figure S1A). These results suggest that ZmBES1/BZR1-5 may play an important role in stress responses by regulating gene expression of its downstream targets.

In fact, a previous study has shown that the *bes1-D* mutant (En2 background), a monogenic semi-dominant mutation, displays constitutive BR responses through suppressing *bri1* phenotypes [17]. The *bes1-D* mutant is more sensitive to drought stress than En2 and Col-0 wild types, indicating that BES1 positively regulates drought tolerance [34]. In the present study, the expression of *ZmBES1/BZR1-5* enhanced the salt and drought tolerance in transgenic *Arabidopsis* in a *bes1-D* background. The transgenic lines showed higher fresh weight, developed roots as well as lower MDA content and REL in transgenic *Arabidopsis* (Figure 3, Figure 4 and Figure 5), probably due to BES1/BZR1s regulating target gene expression in root apex cells, resulting in the control of root elongation and response to warmth [47,48]. As is well known, plants can respond to soilborne stresses, including salinity and water deficit, by adjusting the root system architecture [7,8]. Likewise, BES1/BZR1s target miRNA396d to regulate plant height through the gibberellin signaling pathway in rice [49]. The abiotic stresses could accelerate accumulation of reactive oxygen species (ROS) that catalyze lipid peroxidation of polyunsaturated fatty acids to produce MDA, resulting in damage to the cell membrane [50]. Hence, the MDA content and REL are widely used as biomarkers to evaluate plant tolerance to abiotic stresses. The lower MDA content and REL in transgenic *Arabidopsis* when compared to the untransformed *bes1-D* mutant (Figure 5) could be due to the regulation of *ZmBES1/BZR1-5* expression, resulting in increased glutathione S-transferase gene (*GST*) and peroxidase gene (*POD*) expression and, hence, scavenging of drought-induced superoxide anions (O_2_^-^) [32]. The expression of *ZmBES1/BZR1-5* also decreased the ABA sensitivity of the transgenic lines (Figure 3), suggesting that it positively regulates the ABA response. It had been shown that the expression of the *BES1/BZR1* gene was significantly regulated by ABA in maize and *Brassica rapa* [23,44], and ABA enhanced phosphorylation of the BES1/BZR1 protein in *Arabidopsis* [51]. 

Specifically, the BES1/BZR1s possessed one highly conserved basic helix–loop–helix (bHLH) domain in the N terminal, which contributes to the binding of the promoter of target genes [18,22]. Besides the bHLH domain, ZmBES1/BZR1-5 also contained one β-amylase (BAM) domain, which functions as a transcription factor and is critical for the bHLH-DNA recognition and transcriptional activation [23,52,53]. Proteins that harbor the BAM domain have been reported to be involved in plant growth and drought stress [52,53,54,55], indicating that ZmBES1/BZR1-5 could alter the expression of downstream genes. In the common DEGs in transgenic lines identified by RNA-seq, multiple abiotic stress-related genes are potentially regulated by ZmBES1/BZR1-5 through the binding of E-box in their promoters (Figure 6 and Figure 8). For example, ZmBES1/BZR1-5 directly acts on the rare cold-inducible 2a gene (*RCI2A*, AT3G05880) (Figure 6), and can be induced by salt, dehydration and ABA stress, resulting in improvement of salt tolerance [42,43].

Taken together, our work reveals that the expression of the *ZmBES1/BZR1-5* gene decreases ABA sensitivity and enhances osmotic stress tolerance in transgenic *Arabidopsis* by regulating the expression of multiple stress-related genes. Our study provides new insights into understanding the function and mechanism of the response of BES1/BZR1 family members to abiotic stress in plants. 

## 4. Materials and Methods

### 4.1. Plant Materials and Growth Conditions

The seeds of maize inbred line B73 were germinated in a petri dish, then transplanted into a plastic mesh grid for hydroponic culture under 16 h light at 28 °C/8 h dark at 25 °C and used for osmotic treatments. Tobacco (*Nicotiana benthamiana*) were cultured in a growth chamber under 10 h light at 20 °C 14 h dark at 26 °C with 60–70% relative humidity. The *Arabidopsis* mutant of the *BES1* gene (*bes1-D*, CS65988, *Enkheim-2* ecotype background) was grown in a green house at 22 °C and 60–70% relative humidity under a 10 h light/14 h dark photoperiod. 

### 4.2. Material Preparation and Expression Analysis 

At the three-leaf stage, the B73 seedlings of the same size were divided into two groups for osmotic stress treatment, including salt and PEG treatments. For the salt treatment, one group of seedlings was subjected to a 250 mM NaCl solution with three replicates. For the PEG treatment, another group of seedlings was treated with 16% (w/v) PEG-6000 solution with three replicates. At 0 (control), 1, 3, 6, 9, 12 and 24 h of treatment, the leaf and roots were sampled, and immediately ground in liquid nitrogen for RNA extraction. Total RNA was extracted from all samples using the RNAiso plus kit (TaKaRa, Dalian, China), then quantified using NanoDrop^TM^ One^C^ (ThermoScientific, Waltham, MA, USA) and reverse transcribed into cDNA using the PrimeScript^TM^ reagent kit (TaKaRa) according to the manufacturer’s instruction.

A pair of specific primers (qF1: 5′-CCTGGCAGGTCATCAACGC-3′; qR1: 5′-AAGGTGCAGAGCTCCGAAAG-3′) was designed and synthesized at Sangon biotech (Shanghai, China) and used to amplify a 191 bp fragment of the *ZmBES1/BZR1-5* gene. Another set of specific primers (qF2: 5′-CCATCACTGCCACACAGAAAAC-3′; qR2: 5′-AGGAACACGGAAGGACATACCAG-3′) was designed, synthesized and used to amplify the 171 bp fragment of the *ZmGAPDH* gene as internal reference. The qRT-PCR was performed using 2 × SYBR^®^ PremixEx Taq^TM^ II (Takara, Dalian) in the CFX96^TM^ Real-Time System (Bio-Rad, USA). The two-step temperature cycle was as follows: 95 °C for 30 s; 39 cycles of 95 °C for 5 s and 58 °C for 30 s. At the end of the last cycle, the temperature was increased to 95 °C at 0.5 °C/s, so that a melting curve could be calculated and used to differentiate specific and non-specific amplicons. The relative expression level was calculated and normalized using the 2^−ΔΔCT^ method of the CFX Manger™ software version 2.0 (Bio-Rad, Berkeley, CA, USA).

### 4.3. Subcellular Localization of ZmBES1/BZR1-5 in Tobacco

The open reading frame (ORF) of the *ZmBES1/BZR1-5* gene without a stop codon was amplified by the specific primer F3: 5′-ACGCGTCGACATGAAGCACCCGCTGCACCG-3′ and R3: 5′-GGACTAGTACCTTCCCCATTCTGGGGAGCC-3′ (the underlined bases are the *Sal*I and *Spe*I site, respectively). The amplified ORF fragment and pCAMBIA2300-*35S*-*eGFP* plasmid was digested by endonuclease digestion with *Sal*I/*Spe*I, and then mixed for ligation using T4 DNA ligase to generate the *ZmBES1/BZR1-5* fusion *eGFP* construct named *35S*-*ZmBES1/BZR1-5*-*eGFP*. The *35S*-*ZmBES1/BZR1-5*-*eGFP* and *35S*-*eGFP* plasmid (control) were transformed into the *Agrobacterium tumefaciens* strain GV3101, then used for transient expression in tobacco. The one-month-old seedlings were used for *Agrobacterium*-mediated infiltration. *A. tumefaciens* harboring *35S*-*ZmBES1/BZR1-5*-*eGFP* and *35S*-*eGFP* were cultured overnight at 28 °C and centrifuged for 10 minutes at 5000 rpm. The *A. tumefacien* cultures were resuspended using the buffer solution with 50 mM MES (pH 5.6), 2 mM Na_3_PO_4_ and 5% glucose, and immediately infiltrated into the tobacco leaf. The seedlings were grown in a growth chamber under optimal conditions for 3 days. Subsequently, the three-leaf sample infiltrated by *A. tumefaciens* were sampled and GFP fluorescence was observed using the confocal microscope LSM800 (Carl Zeiss, Oberkochen, Germany). 

### 4.4. Transformation of Arabidopsis

*Arabidopsis bes1-D* plants at the flowering stage were used for *Agrobacterium*-mediated transformation according to the floral-dip method. As described by Desfeux et al. [56] with minor modifications, a 200 mL overnight culture of *A. tumefaciens* harboring *35S*-*ZmBES1/BZR1-5*-*eGFP* was centrifuged for 10 minutes at 5000 rpm and resuspended using a buffer solution containing 5% (w/v) sucrose, 10 mM MgCl_2_ and 0.1% Silwet L-77 surfactant. The flower buds of the *Arabidopsis* plants were soaked in the above solution for 1 min, then covered with a black plastic dome and kept in the dark for 24 h. After transformation, the plants were cultured under optimal conditions. As described by Yu et al. [57], the T_1_ seeds were harvested, surface-sterilized using 75% ethanol for 1 min and 10% NaClO for 10 min, washed three times with sterile distilled water, resuspended in 300 μL 0.1% sterilized agar and plated onto 1/2× MS medium plates with 50 mg/L kanamycin (Sigma, Saint Louis, MO, USA) for screening of the transformants. The kanamycin-resistant seedlings were used for GFP fluorescence imaging using the confocal microscope LSM800 (Carl Zeiss, Oberkochen, Germany) with the *bes1-D* mutant used as a control. The T_0_ plants with GFP fluorescence were used to establish the T_2_ generation via self-pollination. The T_2_ plants that segregated in a 3:1 ratio to resistance/susceptibility of kanamycin, corresponding to a single copy of the *ZmBES1/BZR1-5* gene having been inserted into the *Arabidopsis* genome, were self-pollinated to generate T_3_. The homozygous lines without segregation in T_3_ were selected, and two independent transformants, including the L1 and L6 lines with GFP fluorescence in the nucleus, were used for further study (Figure S1). 

### 4.5. Determination of Root Length and Fresh Weight

The seeds of the *bes1-D* mutant and T_3_ transgenic lines were surfaced-sterilized and were placed on 1/2× MS medium plates with either 150 mM NaCl, 300 mM mannitol or 30 uM ABA, with three replicates for each line and condition. The plates were incubated for 2 days at 4 °C in the dark, and vertically cultured under 14 h light/10 h dark for three weeks. The root length and fresh weights of 25 seedlings were monitored for three replicates of each line. 

### 4.6. Salt and Drought Treatment

Three-week-old *Arabidopsis* seedlings were subject to salt treatment according to the methods of Yu et al. [57] with minor modifications, which involved exposure to water solution supplemented with 250 mM NaCl twice with an interval of 3 days between exposures. The phenotype of each line was monitored after 10 and 15 days of salt treatment. For the drought treatment, the two-week-old seedlings were kept under water deprivation for two weeks, then re-watered with a recovery time of one week, and photographed for phenotyping.

### 4.7. Determination of MDA Content and REL

After 3 days of NaCl treatment and 7 days of drought treatment, ten leaves were collected from each line and used to measure MDA content and REL. MDA was detected using the micro MDA assay kit (Solarbio, Bejing, China) according to the manufacturer’s instructions. The fresh weight (W) was determined for leaf samples, followed by homogenization in 1 mL extract buffer in an ice bath and centrifugation at 8000× *g* for 10 min at 4 °C. The supernatant was used to measure absorbance of A532, A600 and A450 with NanoDrop^TM^ One^C^ (Thermo Scientific, Waltham, MA, USA). The MDA content was calculated using the following formula: MDA content (nmol g^−1^) = 5 × (12.9 × (A532 − A600) − 2.58 × A450) ÷ W.

The REL was measured as described by Yu et al. [57] with minor modifications. Leaves were sampled, rinsed with deionized water and dried with filter paper. A 0.3 g sample of leaf was placed into a 50 ml tube with 20 ml deionized water for 5 h. The conductivity (C_1_) was measured with a conductivity meter model DDS-11 (Biocotek, Ningbo, China). Subsequently, the samples were boiled for 15 minutes, cooled at room temperature and used to measure conductivity (C_2_) again. REL was calculated using the following formula: REL = (C_1_/C_2_) × 100%.

### 4.8. RNA Extraction and RNA Sequencing

Two-week-old seedlings were collected from 1/2× MS plates and used for RNA-seq with three biological replicates. The total RNA was extracted using an RNA extractor kit (Sangon, China) according to the manufacturer’s protocol, and treated with RNase-free DNase I to remove genomic DNA contamination. The quality of RNA was assessed using an RNA HS assay kit (Thermo Scientific), quantified with Qubit2.0 (Thermo Scientific). The qualified RNA was used for sequencing and library construction using VAHTSTM mRNA-seq V2 Library Prep Kit for Illumina® following the manufacturer’s recommendations. The sequencing of the library was performed on an Illumina HiSeq3000 system at Sangon biotech (China). After evaluating and filtering raw data using FastQC (version 0.11.2) and Trimmomatic (version 0.36), the clean data were mapped to the *Arabidopsis* genome (TAIR10) by hisat2 [58] and used to assemble transcripts and to calculate the expression of genes using StringTie [59]. The DEGs were analyzed by Deseq2 [60]. Genes were considered as significantly differentially expressed at a *p*-value < 0.05 and |FoldChange| > 2. The GO enrichment analyses and KEGG pathway functional enrichment analysis of the DEGs were performed using KOBAS (http://kobas.cbi.pku.edu.cn/anno_iden.php) [61].

### 4.9. Yeast One Hybrid Assay

To validate the ability of ZmBES1/BZR1-5 to bind to DNA, a Y1H assay was performed as in [26] with minor modifications, a construct containing four copies of the E-box (4 × E-box: 5′-CGCATTTGGGGGCAGATGGGCTCACATGATAACAAGTGGT-3′, with the underlined bases indicating the E-box elements) was designed, synthesized and cloned into the pAbAi vector to generate pAbAi−4 × E-box. The −795 to −1246 bp of *RCI2A* promoter was amplified from B73 genomic DNA using the specific primers F4: 5′-GCATCGAT AGTTTTAACTTTACATTTGTTATTT-3′ and R4: 5′-AGCTCGAG ATAGTCGATTCAACTGATTCATGTC-3′, and then cloned into the pAbAi vector to generate pAbAi_−795_ to _−1246_*RCI2A*. The pAbAi−4 × E-box and pAbAi_−795_ to _−1246_*RCI2A* plasmid were linearized with *Bst*BI, transformed into the Y1H gold yeast strain and screened on SD/−Ura plates with 400 ng/ml AbA. The ORF of the *ZmBES1/BZR1-5* gene was amplified using the specific primers F5: 5′-GCATCGATATGAAGCACCCGCTGCACCGCG-3′ and R5: 5′-AGCTCGAGTCAACCTTCCCCATTCTGGGGA-3′ (the underlined bases indicate the *Cla*I and *Xho*I site, respectively) and cloned into the *Cla*I/*Xho*I site of pGADT7 vector to create pGADT7−*ZmBES1/BZR1-5*. The pGADT7−*ZmBES1/BZR1-5* and empty pGADT7 plasmid was transformed into the Y1H gold yeast with pAbAi−4 × E-box, pAbAi_−795_ to _−1246_*RCI2A*, respectively. After transformation, the yeast cultures were streaked onto SD/−Leu plates with 400 ng/ml AbA, and incubated for 3 days at 30 °C before being photographed.

### 4.10. Statistical Analysis

The data are presented as the mean values ± standard deviation (SD). Statistical analyses were performed by Microsoft Excel 2017 and SPSS 17.0 software based on Student’s *t* tests to determine whether the difference between the treatment or transgenic lines and the control were considered significant.

## Supplementary Materials

The following are available online at https://www.mdpi.com/1422-0067/21/3/996/s1, Figure S1: Transgenic lines harboring *ZmBES1/BZR1-5-GFP* were used for further study, Table S1: DEGs that are involved in stress or stimulus response, Table S2: The number of E-box or BRRE elements in promoter regions of 30 stress-related DEGs.
