# Supplementary material for: Maize ZmBES1/BZR1-5 Decreases ABA Sensitivity and Confers Tolerance to Osmotic Stress in Transgenic Arabidopsis
Source: Int J Mol Sci. 2020 Feb 3;21(3):996. doi: 10.3390/ijms21030996 (PMC7036971; doi:10.3390/ijms21030996)
Supplement: Supplementary file 1 [file ijms-21-00996-s001.zip › ijms-679942 Supplimentary figure.pdf]

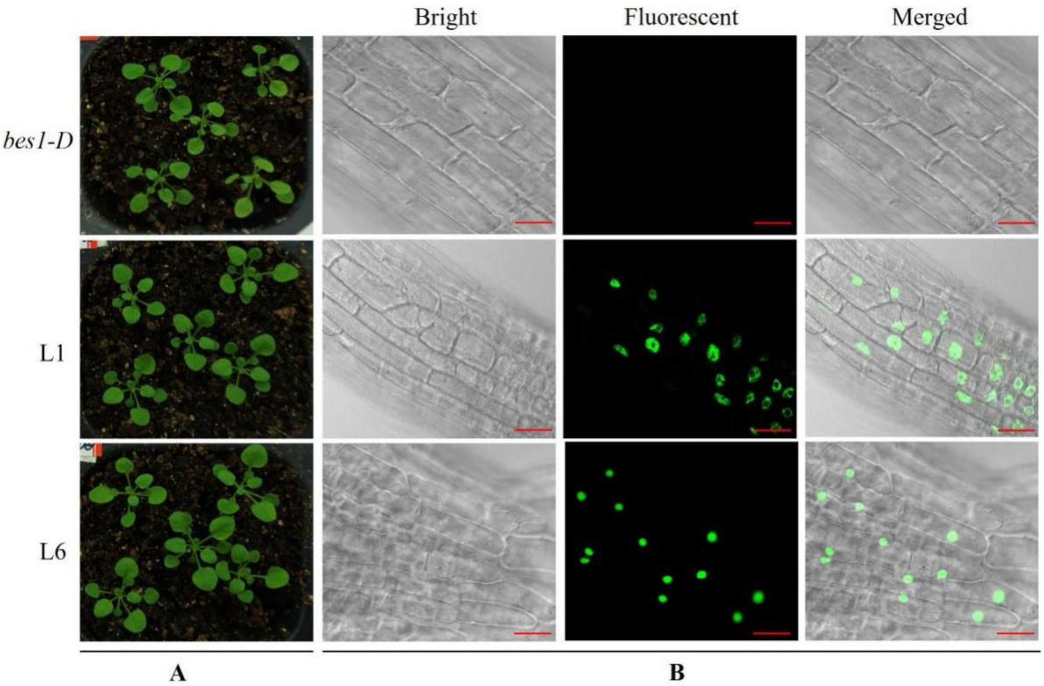

Figure S1. Transgenic lines harboring ZmBES1/BZR1-5-GFP were used for further study. (**A**) The phenotype of every line under standard growth condition. (**B**) The GFP fluorescence signal test in the seedlings root. *bes1-D*, L1 and L6 represents untransformed mutant and homozygous T_3 lines, respectively.
